# Protocol for the development of NHMRC-endorsed guidelines for extracorporeal membrane oxygenation using GRADE methodology^☆^

**DOI:** 10.1016/j.ccrj.2024.11.002

**Published:** 2025-03-01

**Authors:** Sally F. Newman, Zachary Munn, Craig French, Hergen Buscher, Daniel Thomas Chung, Myles Smith, Madeline Wilkinson, Priya Nair

**Affiliations:** a Department of Intensive Care Medicine, St Vincent's Hospital, Darlinghurst, NSW, Australia; b School of Clinical Medicine, University of New South Wales, Kensington, NSW, Australia; c School of Public Health and Preventative Medicine, Monash University, VIC, Australia; d Te Puna Wai Ora, Southern Critical Care, Dunedin Hospital, Te Whatu Ora, New Zealand; e University of Otago, Dunedin, Otago, New Zealand; f University of Notre Dame, Darlinghurst, NSW, Australia; g Health Evidence Synthesis, Recommendations and Impact (HESRI), School of Public Health, The University of Adelaide, SA, Australia; h Department of Intensive Care Medicine, Western Health Services, Melbourne, VIC, Australia

**Keywords:** Practice guideline, Extracorporeal membrane oxygenation, Evidenced-based medicine, Guideline protocol

## Abstract

**Introduction:**

The last 15 years have seen a rapid expansion in the use of extracorporeal life support. ECMO has evolved from a rescue treatment available in a few expert centres to an organ support modality for many forms of severe respiratory or cardiovascular failure. There is currently wide variation around the indications for, management of, and systems to support the practice of ECMO. There are few available guidelines on this topic; most have limitations and are not readily generalisable to the Australian or New Zealand healthcare systems.

**Methods and analysis:**

This article aims to describe the processes that will be used to produce evidence-based guidelines on the use of ECMO in Australia and New Zealand. The protocol is informed by the National Health and Medical Research Council (NHMRC) Guidelines for Guidelines, and the Grading of Recommendations, Assessment, Development and Evaluation (GRADE) framework.

Analysis of available evidence on the identified questions follows a three-phase approach. Firstly, published guidelines will be identified and an assessment of their relevance, methodology and validity carried out. If there are no guidelines on the topic, the second step involves a search and evaluation of systematic reviews. Lastly, a de-novo systematic analysis of primary literature will be undertaken where no systematic reviews are available. The development process will be conducted using the GRADEpro and Covidence software for de novo systematic reviews.

**Dissemination:**

The guideline will be published in peer-reviewed journals and summaries will be provided to end-users via the GRADEpro GDT application.

## 1 Introduction

Extracorporeal membrane oxygenation (ECMO) provides cardiac and/or respiratory support to patients with severe organ failure. While ECMO support has been used in paediatric populations for some decades its use in adults has increased, particularly over the last 15 years. This expansion is described in the Extracorporeal Life Support Organisation (ELSO) registry, International Summary (Supplementary File 1):^1^ 8778 ECMO runs are reported in the first 7 months of 2024.^1^ The COVID-19 pandemic increased worldwide utilisation of ECMO in severe acute respiratory syndrome (SARS), with 11,787 patients managed on ECMO and an in-hospital mortality rate of 47 %.^2^ This rapid increase in the use of ECMO occurs in the absence of high-quality data to inform this invasive, and resource-intensive intervention. Evidence-based guidelines are important to facilitate the safe and effective application of medical interventions. Recognising this gap, the ACTIONS-CRE (Centre for Research Excellence in Advanced Cardiorespiratory Therapies Improving OrgaN Support) at University of Queensland commissioned the ‘National ECMO Guidelines Project’. Clinical guidance for the use of ECMO is currently largely based on expert consensus, case series and cohort analyses; only a handful of randomised controlled trials have been performed.^3^^,^^4^ The number of ECMO-related publications has also increased dramatically, making it difficult for ECMO providers to keep up to date and evaluate this expanding pool of literature efficiently.^4^

A survey of twenty-three ECMO-providing sites across Australia showed that 82 % were guided by local clinical practice guidelines: these varied significantly, specifically in patient selection, the reporting of outcomes and training and credentialing practices.^5^ The current ECMO guidelines in Australia and New Zealand exist at a local and state level and based on physiological principles and clinical experience.

The emphasis of the Council of Australian Governments (COAG) placed on improving healthcare safety and quality aligns with the broader global goal of enhancing patient outcomes and optimising healthcare delivery.^6^ Creating guidelines using rigorous methodology is a key strategy to achieve these goals. The standard of Clinical Governance states that healthcare providers are responsible for supporting and improving healthcare reliability, safety and quality. These guidelines will help reduce variations in ECMO care, potentially improving patient outcomes, and ensure appropriate patient selection and timing. The guidelines will also provide direction for ECMO training and education for healthcare professionals. They may also aid efficient and cost-effective resource allocation. The objective of this article is to describe the protocol for the development of these guidelines.

## 2 Methods

These guidelines will be developed following the National Health and Medical Research Council (NHMRC) Guidelines for Guidelines framework.^7^ This includes:

(a) Planning

The Guideline Development Group (GDG) will include experts from the medical, nursing and allied health fields, with representation from the relevant craft groups of intensive care medicine, cardiothoracic surgery, anaesthetic and perfusion disciplines. This will include specialists from non-ECMO centres. In addition, a research methodologist will provide guidance on the GRADE methodology application. Members on the GDG will include representation across the national and international ECMO communities. All jurisdictions across Australia and New Zealand will be represented in this group. Vitally, there will be active consumer participation from survivors of ECMO. Involvement of these individuals will ensure that the guidelines are consumer focused. This is in keeping with the Patient-Centred Outcomes Research Institute recommendations which have emphasised the importance of involving patients and other stakeholders in all aspects of the research process.^8^ A project manager with experience in guideline development and clinical knowledge of ECMO will facilitate communication, collaboration and project progression. The project manager along with two or three members from the GDG will form the ‘Evidence Team’. Members of the evidence team will vary depending on the Population-Intervention-Comparison-Outcome (PICO) question being addressed. Consistency and quality of the process across all the PICO questions will be ensured and facilitated by the project manager. The expectations of participation will be outlined in the terms of reference document and shared with members. Conflicts of Interest (COI) will be clearly defined and made known to all committee members, and they will be prompted to update their disclosures regularly as circumstances change. Any potential or perceived COI will be discussed and managed on a case-by-case basis.

(b) Development

### 2.1 Framing and prioritising Population-Intervention-Comparison-Outcome questions

Guideline Development Group members will be invited to submit questions that they consider clinically important in the PICO format for binary comparisons or free form for open-ended questions. These submissions will be made via the GRADEpro® GDT software. This software provides a platform for the drafting, brainstorming and prioritising of potential clinical questions. To limit the number of questions included in the guideline the GDG will rank the questions on a scale from 1 to 9 (7–9 – critical; 4 to 6 – important; 1 to 3 – of limited importance). Only questions rated critical will be answered with recommendations. The priority ranking process will be repeated until the total number of questions reaches fifteen or less. The GDG concluded that 15 questions would be reasonable to address within this project.

### 2.2 Selecting and rating importance of outcomes

For each PICO, outcomes that are important or critical to patients for decision making will be listed. The consumer representatives on the panel play a key role in guiding this step. Accordingly, using the software's voting system, the panel will decide whether the outcomes are critical, important but not critical or of limited importance.

### 2.3 Identifying and synthesising evidence

**Fig. 1:**
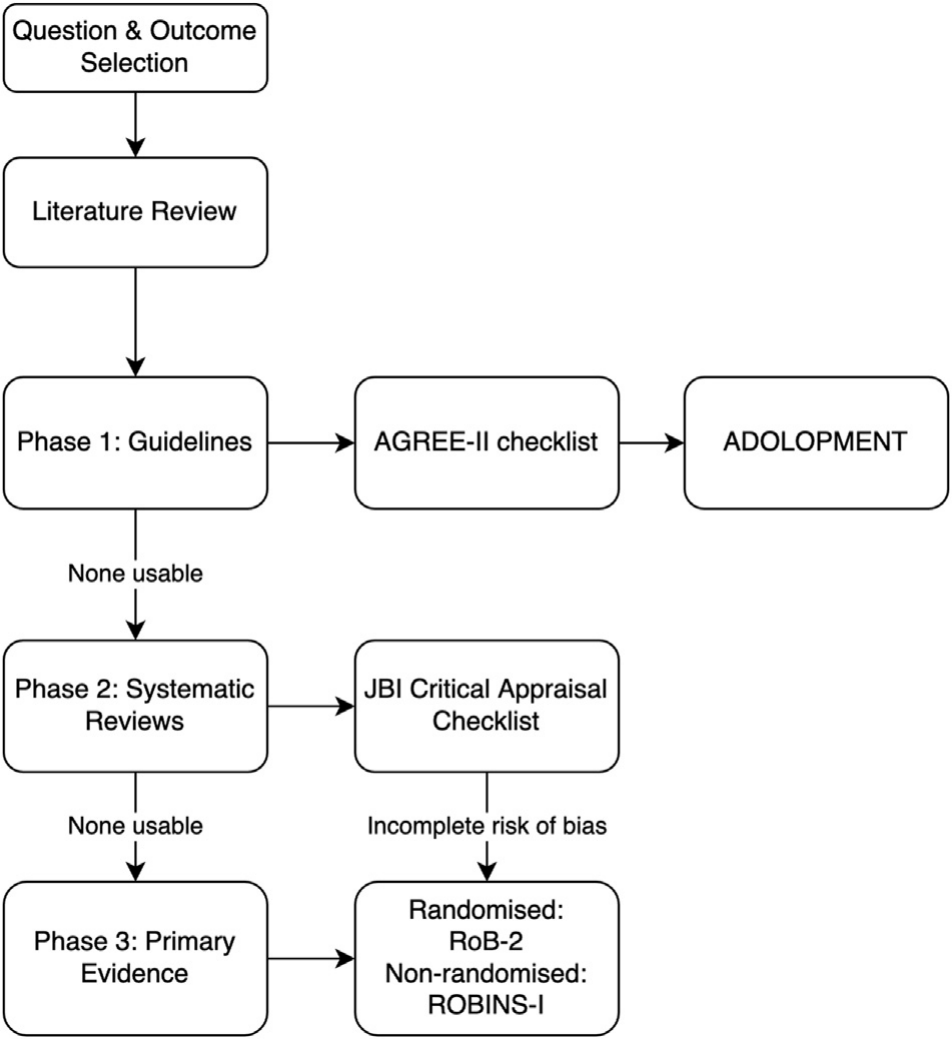
Schematic view of 3-phased workflow approach.

For each question, the evidence team will examine the possibility of adopting or adapting existing guideline recommendations. The utilisation of methodology that combines the advantages of adoption, adaptation, and de novo development of recommendations (known as “GRADE-ADOLOPMENT”)^9^ in a 3-phase workflow (Fig. 1) will be applied. In **phase one**, guideline portals will be searched for existing guidelines. Eight guideline portals will be searched, including GIN Library,^10^ ECRI Guidelines Trust,^11^ Database of GRADE Evidence to Decisions (EtD) and Guidelines,^12^ MAGICapp,^13^ BIGG International database of GRADE guidelines,^14^ National Institute for Health and Care Excellence guidelines,^15^ ELSO Guidelines^16^ and TRIP database.^17^

If available, guidelines will be assessed for relevance, currency, trustworthiness, access to evidence, feasibility and acceptability.

To consider adopting or adapting an existing guideline, the presence of the following criteria will be considered and evaluated:

(i) The questions on which the guideline and reviews of evidence are based.
(ii) Evidence tables that summarise the characteristics of the studies included.
(iii) A Summary of Findings (SoF) table containing the main findings of an evidence review based on a specific question and selection of outcomes.
(iv) Evidence to Decision (EtD) framework that structures the formulation of recommendations, including detailed information on decisions made regarding questions, criteria, evidence, judgements and conclusions.
(v) The guideline's recommendations for policy or practice.
(vi) Content in the guideline that supports the recommendations, such as tables, figures, practice points or other text.

Guideline appraisal will determine quality and trustworthiness of the evidence presented utilising the AGREE II tool.^18^

If the above criteria are not met and no adoption or adaptation is possible, a **phase two** search of existing systematic reviews will be performed. Two databases, Epistemonikos^19^ and PROSPERO,^20^ will be utilised to search for systematic reviews. Systematic reviews will be searched, reviewed and included if they cover the same population, intervention and outcomes as the chosen topic. Critical appraisal of the systematic review using the Joanna Briggs checklist,^21^ will determine quality and trustworthiness. If an appropriate systematic review is found, the reviews results will be assessed for all critical and important outcomes and used to populate a GRADE Summary of Findings (SoF) table.^22^

Where systematic reviews do not cover relevant PICO questions, or do not include RCTs completed within the past five years, a de-novo systematic review will be attempted in **phase three**. Primary literature searches will be completed using MEDLINE,^23^ EMBASE^24^ and CENTRAL.^25^ Due to a paucity of randomised control studies in this area, observational studies will be included. Consecutive case series will be considered if they include ≥10 subjects. Randomised controlled trials will be assessed for their risk of bias using the RoB-2 tool^26^ and non-randomised studies will be assessed using the ROBINS-I tool.^27^ Two team members will independently conduct literature screening, risk of bias assessment and data extraction. Discrepancies will be resolved with a third team member. The GDG will consider the appropriate use of project resources for each question while progressing through the three phases. A schematic view of this process taken from the GRADE handbook is available in Supplementary file 2.^28^

Evidence searches of all phases will be guided by a research librarian. The web-based software platform Covidence®^29^ will be used for reference management, screening, selection and data extraction and to streamline the review process.

### 2.4 Assessing the certainty of the evidence

The certainty of evidence will be assessed using the GRADE approach. The factors that reduce the quality of evidence when using GRADE are:

(a) risk of bias
(b) inconsistency of the results - consistency of results across all studies
(c) indirectness of the evidence - relevance of the evidence to the specific clinical question
(d) imprecision - the precision of estimates and confidence intervals and where they lie in relation to important decision-making thresholds and whether the sample size is sufficient to draw a reliable conclusion and
(e) publication bias will be considered.

Given the context of this guideline and the literature available, the factors generally considered to increase the quality of evidence (large magnitude of effect, dose–response gradient and effect of plausible residual confounding) will be considered, however, unlikely to be encountered.

### 2.5 Evidence to decision framework

This is an integral part of the GRADE methodology that provides a structured and transparent process to move from the evidence to recommendations. The key components enumerate and identify all factors relevant to the decision-making process, including benefits and harms, values and preferences, resource implications, equity, acceptability and feasibility.^28^

### 2.6 Generating a recommendation

Synthesis of all the information gathered within the EtD framework will be presented to the GDG and culminate in an explicit recommendation. The recommendations will be categorised as strong or weak, with clear justifications provided. The process involves voting and judgements by the GDG within the GRADEPro® software considering-

(a) whether the problem is a priority
(b) how substantial the desirable effects are
(c) how substantial the anticipated undesirable effects are
(d) the overall certainty of evidence
(e) whether there is important uncertainly or variability in how much the main outcomes are valued
(f) whether the balance between desirable and undesirable effects favours the intervention or the comparison (g) the resource requirements
(h) cost effectiveness
(i) acceptability to stakeholders
(j) impact on health equity
(k) feasibility of delivery of the intervention.

The recommendations for or against the intervention will then be categorised as strong or weak by objectively reviewing the summary of judgements table that is generated by the software at the end of this process.

It is anticipated that the process will generate questions involving comparison of multiple interventions in a population, for instance “What is the best strategy?” where multiple strategies exist in the literature. For these overarching questions, the approach suggested by Piggott et al.^30^ will be used to group interventions and rate them across each of the EtD criteria. The GRADEPro software tool designed for this process will be used. This should allow users of the guideline to understand the competing factors in any recommendation on a question with multiple possible options.

(c) Review

On completion of the recommendation, where there are identified international experts relevant to a specific PICO question, their opinion will be requested. In addition, endorsement from relevant local peak bodies such as medical colleges and specialty societies will be sought. Considering the target audience of the guideline, the GDG will seek to engage stakeholder organisations such as The College of Intensive Care Medicine of Australia and New Zealand (CICM), The Australian and New Zealand Intensive Care Society (ANZICS), Australian College of Critical Care Nurses (ACCCN), Australia New Zealand College of Perfusionists (ANZCP), Australia New Zealand Society of Cardiothoracic Surgeons (ANZCTS) and the NHMRC for endorsement.

(d) Implementation

The GDG will ensure these guidelines provide practical, feasible, and relevant recommendations to assist end users with ease of uptake.^7^ Table 1 illustrates six principles underpinning implementation and how we will adapt them within this project. This dissemination plan includes access across multiple channels, such as digital options, summaries, conference abstracts and peer-reviewed publications. GRADEpro® software^31^ provides options to generate links to inform target audiences, healthcare providers, policymakers and consumers through online platforms. Patient information pamphlets will be in plain English and available via the Internet.

**Table 1:** Principles of implementation and examples of how they will be applied within this project.

Principles of Implementation	Examples of application
Stakeholders: Engage a wide variety	Consumers, doctors, nurses, physiotherapists, representative of government policymaker boards will be engaged and included
Trustworthiness: Transparent methods	GRADEpro® GDT software and development of a full technical report
Judgement: Judgements used to balance evidence, benefits and harms	EtD frameworks will be used
Feasibility: Assess local applicability	EtD framework considers feasibility GDG members from across jurisdictions
Clarity of message	Clear and actionable recommendations with justifications
Appropriate formats: Dissemination platforms	End user specific information with appropriate language Clinicians, patients and family, policymakers

EtD, evidence to decision; GDG, guideline development group.

## 3 Discussion

ECMO is a resource-intensive, invasive intervention whose use is increasing. There is an unmet need for ECMO guidelines that follow an internationally accepted, transparent framework and review process in Australia and New Zealand. The methodology for this guideline includes a comprehensive and rigorous three-phase search to identify international guidelines, systematic reviews or primary literature: to our knowledge no guidance utilising this approach exists in the field of ECMO. The assessment of certainty of evidence and strength of recommendations will be undertaken using GRADE methodology. A guideline development group comprising broad clinical and methodological expertise and including consumer representation will enhance the relevance of the guidelines.

The guidelines will only address the prioritised PICO questions which will not cover the whole spectrum of clinical practice in the field of ECMO.

Finally, areas lacking in research will be highlighted during the exhaustive guideline development process, paving the way for future local ECMO research.

## CRediT authorship contribution statement

**Sally Newman**: Conceptualisation, methodology, writing – original draft, incorporated feedback, finalised the manuscript and project administration. **Zachary Munn**: methodology, review and editing, **Craig French:** review and editing **Priya Nair:** Conceptualisation, writing – review & editing, funding acquisition, project administration. **Hergen Buscher**: Conceptualisation, writing – review & editing, project administration. **Myles Smith:** Conceptualisation, writing – review & editing, project administration. **Madeline Wilkinson**: conceptualisation, methodology. **Daniel Chung:** review and editing.

## Funding statement

The work was funded by the ACTIONS-CRE (Centre for Research Excellence in Advanced Cardiorespiratory Therapies Improving OrgaN Support) at the 10.13039/501100001794University of Queensland and CRE-ICU (A CRE to Transform Outcomes after Critical Illness, GNT 1196602) at the ANZIC RC. The funders had no part in the study design, conduct or data analysis and did not have any authority over these activities.

## Conflict of interest

The authors declare the following financial interests/personal relationships that may be considered as potential competing interests: Sally Newman reports financial support was provided by Monash University. If there are other authors, they declare that they have no known competing financial interests or personal relationships that could have appeared to influence the work reported in this article.
