# Supplementary material for: Protocol for the development of NHMRC-endorsed guidelines for extracorporeal membrane oxygenation using GRADE methodology
Source: Crit Care Resusc. 2025 Mar 1;27(1):100093. doi: 10.1016/j.ccrj.2024.11.002 (PMC11915137; doi:10.1016/j.ccrj.2024.11.002)
Supplement: Multimedia component 1 [file mmc1.docx]

Supplementary File 1.


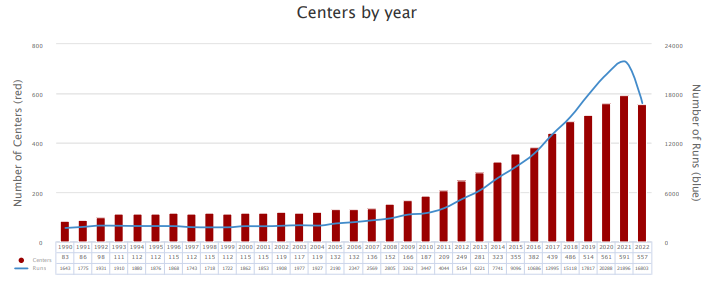


Extracorporeal Life Support Organisation (ELSO) International Summary of 2022 shows summary of ECMO-supported patients from the ELSO Registry over three decades.

Supplementary File 2.


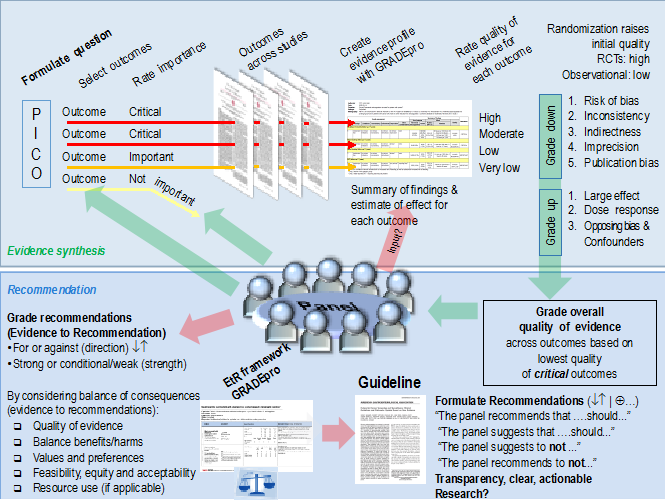


A schematic view of the GRADE approach for synthesising evidence and developing recommendations taken from the GRADE Handbook.
